# Assessment of Differences in Oral Health Knowledge, Attitudes, and Behavior Among Preclinical and Clinical Dental Students

**DOI:** 10.7759/cureus.52509

**Published:** 2024-01-18

**Authors:** Sumit Mohan, Gaurav Kumar, Butta Viswanath, Harsh Priyank

**Affiliations:** 1 Conservative Dentistry and Endodontics, Dental College, Rajendra Institute of Medical Sciences, Ranchi, IND; 2 Conservative, Endodontics and Aesthetic Dentistry, Dental College, Rajendra Institute of Medical Sciences, Ranchi, IND

**Keywords:** knowledge, oral health, dental students, behavior, attitude

## Abstract

Aim

To assess and compare differences in oral health knowledge, attitudes, and behavior among preclinical and clinical dental students of Dental Institute, Rajendra Institute of Medical Sciences, Ranchi, Jharkhand, India.

Material and methods

A total of 175 students responded to a total of 37 questions regarding their knowledge, attitudes, and behavior regarding dentistry and oral health. The mean percentage scores and standard deviation were calculated to assess the relation between knowledge, attitude, and behavior.

Results

It was observed that the students in the clinical phase had significantly better knowledge and attitude towards oral health than preclinical undergraduates. There was no significant difference in mean and SD among clinical and preclinical students in behavior while statistically significant differences were observed in their responses to questions related to knowledge (p = 0.000) and attitude (p = 0.007). Female students had better knowledge than male students (p = 0.029).

Conclusion

Clinical dental students of the institute showed a marginally higher KAP regarding oral health than preclinical students. This might reveal an ineffective transition of the students from the preclinical to the clinical stage. On intergender comparison, the females were better oriented than males towards oral health.

## Introduction

Oral health is a fundamental aspect of overall health and well-being, encompassing not only the teeth and gums but also the broader craniofacial complex. It plays a crucial role in one's quality of life, affecting speech, nutrition, and self-esteem [1]. Dental professionals bear the responsibility of ensuring that individuals receive adequate oral healthcare, which includes prevention, diagnosis, and treatment of oral diseases [2-5]. Consequently, dental education programs serve as the cornerstone for training future dental practitioners, equipping them with the necessary knowledge, attitudes, and behavior required to provide effective oral healthcare [6, 7].

The distinction between preclinical and clinical dental students is pivotal in dental education [8]. Preclinical dental students, typically in their first two years of dental school, are primarily engaged in foundational coursework, including the study of oral anatomy, dental materials, and the basic sciences. In contrast, clinical dental students, often in their third and final years and internship, transition to the clinical phase of their education, where they gain hands-on experience treating patients under the supervision of licensed dental faculty. This phase is essential for integrating theoretical knowledge into practical clinical skills and developing attitudes necessary for patient care. Previous studies assessing the difference in attitude and behavior between preclinical and clinical dental students have reported conflicting results, while some studies found better awareness amongst clinical students [9, 10], others have reported no difference between them [11, 12].

As the demand for dental care continues to rise, it becomes increasingly imperative that dental education programs are not only comprehensive but also attuned to the evolving needs of the healthcare landscape [13]. It is within this context that this study aims to assess and compare oral health knowledge, attitudes, and behavior among preclinical and clinical dental students. The assessment of oral health knowledge, attitudes, and behavior among these two distinct groups of dental students is of paramount importance [14]. It provides insights into the efficacy of dental education programs in equipping students with the requisite competencies and values essential for their future roles as oral healthcare providers [14, 15]. Additionally, understanding the differences and similarities in these aspects between preclinical and clinical dental students can inform curriculum development, teaching strategies, and interventions aimed at improving oral health education [16].

Therefore, this research aims to shed light on the following key question: How do the oral health knowledge, attitude, and practice (KAP) of preclinical dental students compare to that of their clinical counterparts?

## Materials and methods

The present study was conducted among 175 undergraduate dental students of Dental Institute, Rajendra Institute of Medical Sciences, Ranchi after obtaining clearance from the ethics committee vide Memo 47, IEC, RIMS, Ranchi dated 3/6/2020.

Sample size

A purposive sampling technique was employed to select participants. The sample size was determined to be 164, taking into account an 80% confidence interval and a 5% margin of error. However, a total of 175 participants were recruited to account for any attrition.

All 175 participants were invited to participate in this survey comprising 37 questions in English regarding oral health knowledge, behavior, and attitudes. The questions were sourced from previous studies [9-12]. The questionnaire was transformed into an online survey using a web-based survey tool (Google Forms) and the survey link was shared amongst all dental students by their class representatives. A link to the survey was mailed to all students. All the undergraduate students of the institute were included to participate in the study while no exclusion criteria were defined.

Statistical analysis

SPSS statistical software (v.20; SPSS/IBM, Armonk, NY) was used to analyze the data. Since the data had a non-normal distribution according to the Shapiro-Wilk test, non-parametric tests were used. Chi-squared test was used to evaluate variations in responses to each questionnaire item. The Mann-Whitney test was used to compare the mean score of knowledge, attitude, and practice-related items between gender (males and females) and academic level (pre-clinical and clinical). Each correct response was given a score of 1, while erroneous responses were given a value of zero, to create an overall score for the items that tested knowledge, attitude, and behavior (where there were correct answers), giving knowledge-based items a maximum score of 21, attitude-related items a maximum score of 5, and practice-related items a maximum score of 11. A p-value of 0.05 or less was considered statistically significant.

## Results

A total of 175 students participated in the survey. A basic demographic detail of the students is depicted in Table 1. The responses of the participants on questions related to knowledge, attitude, and behavior are charted in Tables 2-4 respectively.

**Table 1:** Demographic Details of the Participants

Academic level	Number of students (n)	Number of Male	Number of Female	Mean (SD) age in years
1^st^ year	57	15	42	21.86 (1.81)
2^nd^ year	49	12	37	22.12 (1.51)
Preclinical (1^st^ & 2^nd^ year)	106	27	79	21.98 (1.65)
3^rd^ year	22	2	20	23.09 (1.57)
4^th^ year	35	9	26	23.86 (1.26)
Internship	12	5	7	24.92 (0.79)
Clinical (3^rd^, 4^th^ years & Internship)	69	16	53	23.8 (1.43)
Total	175	43	132	22.70 (1.81)

**Table 2:** Proportion of participants with acceptable responses to questions on Knowledge

S.No	Questions	Proportion (%) of Preclinical students	Proportion (%) of Clinical students	P value
1	How many sets of dentitions do we have?	89.6	100	0.006
2	How many milk teeth do we have?	96.2	98.6	0.367
3	How many permanent teeth do we have?	99.1	100	0.418
4	What is the purpose of tooth brushing?	89.6	100	0.006
5	What should be the interval for a change of toothbrush?	74.5	78.3	0.573
6	What does plaque mean?	58.5	91.3	0.000
7	What does dental plaque lead to?	84.9	92.8	0.118
8	What does gum bleeding mean?	91.5	98.6	0.050
9	What are the reasons for bleeding gums?	67.9	88.4	0.002
10	What are the methods to prevent bleeding from gums?	70.8	95.7	0.000
11	Do you think that fizzy soft drinks affect the teeth adversely?	98.1	97.1	0.662
12	What is the effect of sweets retention on dentition?	84.9	98.6	0.003
13	What is the reason for tooth decay?	84.9	92.8	0.118
14	Do you think decayed teeth can affect the appearance of a person?	96.2	98.6	0.367
15	What are the methods to prevent dental decay?	84.9	100	0.001
16	What is the effect of fluorides on dentition?	84	94.2	0.042
17	What are the reasons for tooth loss in old age?	34	63.8	0.000
18	Does the health of the mouth and dentition impact the health of the body?	99.1	98.6	0.758
19	Loss of teeth can interfere with speech.	100	98.6	0.214
20	What is the reason for the development of oral cancer?	99.1	100	0.670
21	It is possible to move irregularly placed teeth into the correct position.	95.3	92.8	0.481

**Table 3:** Proportion of participants with acceptable responses to questions on Attitude

S.No	Questions	Proportion (%) of Preclinical students	Proportion (%) of Clinical students	P value
1	Do you think a regular visit to a dentist is necessary?	100	95.7	0.030
2	Do you think the immediate replacement of missing natural teeth by artificial teeth is necessary?	70.8	91.3	0.001
3	Do you think gutkha chewing / smoking is a bad habit?	100	100	-
4	Do you think dentists care only about treatment & not prevention?	94.3	95.7	0.701
5	Do you think that the treatment of toothache is as important as any other organ in the body?	98.1	98.6	0.828

**Table 4:** Proportion of participants with acceptable responses to questions on Behavior

S.No	Questions	Proportion (%) of Preclinical students	Proportion (%) of Clinical students	P value
1	How many times a day do you brush your teeth?	86.8	82.6	0.447
2	When do you brush your teeth?	86.8	81.2	0.313
3	What material do you use for brushing your teeth? (Toothbrush & Toothpaste)	99.1	95.7	0.141
4	If toothpaste, do you use fluoridated toothpaste?	86.8	92.8	0.215
5	What is the reason for you to change the toothbrush?	95.3	98.6	0.246
6	Do you clean your tongue?	99.1	94.2	0.060
7	Do you use any of the oral hygiene aids?	72.6	75.4	0.689
8	If you have visited a dentist, what was the driving factor for your last visit? (Routine visit)	28.3	31.9	0.612
9	How many times a day do you eat sweets?	23.6	18.8	0.457
10	Do you take care of your teeth as much as any other part of your body?	93.4	87	0.149
11	Do you have any habits like Pan chewing, Gutkha chewing, or Cigarette smoking?	98.1	98.6	0.828

There was an insignificant difference in mean and SD among clinical and preclinical students in behavior while we observe statistically significant differences in their responses to questions related to knowledge and attitude. It was observed that the clinical students had superior knowledge and attitude than preclinical undergraduates (Table 5).

**Table 5:** Mean attitude, knowledge, and behavior score (Preclinical and Clinical Students)

Variables	Mean (SD) score of Preclinical students (n = 106)	Mean (SD) score of Clinical students (n = 69)	P value
Knowledge	17.83 (2.299)	19.81 (1.438)	0.000
Attitude	4.63 (0.591)	4.81 (0.550)	0.007
Behavior	8.7 (1.409)	8.57 (1.47)	0.552

On intergender comparison (Table 6), it was observed that female students had better knowledge than males while in questions related to attitude and behavior, their responses were almost similar with insignificant statistical differences.

**Table 6:** Mean attitude, knowledge, and behavior score (Males and Females)

Variables	Mean (SD) score of Males (n = 43)	Mean (SD) score of Females (n = 132)	P value
Knowledge	17.79 (2.73)	18.88 (1.969)	0.029
Attitude	4.81 (0.394)	4.67 (0.626)	0.243
Behavior	8.53 (1.533)	8.68 (1.40)	0.633

## Discussion

The present study was conducted to compare the knowledge, behavior, and attitude among preclinical and clinical undergraduate dental students studying at the Dental Institute, Rajendra Institute of Medical Sciences, Ranchi. Within the limitations of this study, it was observed that there is an insignificant difference between clinical and preclinical students in behavior while we observe statistically significant differences in their responses to questions related to knowledge and attitude. It was observed that the clinical students had superior knowledge and attitude than preclinical undergraduates. Our results are in agreement with the results of studies by Kawamura et al. [17], Tseveenjav et al. [18], and Rong et al. [19]. The difference appears to reflect the variation in the student’s educational level.

On intergender comparison, it was observed that female students had better knowledge than males while in questions related to attitude and behavior, their responses were almost similar with insignificant statistical differences. Our findings were in agreement with the results of Polychronopoulou et al. [20], who inferred that females had better knowledge while Muthu et al. [21], Ostberg et al. [22], and Fukai et al. [23] found that female dental students had better oral health attitudes and take better care of their teeth than their male colleagues. Tseveenjav et al. [18] reported no differences between male and female students of Mongolia in terms of KAP. Students of clinical batches had better knowledge of plaque control and gingivitis and were more aware of the role of diet in the prevention of dental caries.

Results from our study emphasized the fact that both the clinical and preclinical students realized the importance of dental treatment with almost 98% of them saying that treatment of toothache is as important as treatment of any other body part. While all the preclinical students advocated regular checkups from dentists, only 70.8% of them had information regarding the use of artificial teeth. In comparison, 91.3% of the clinical students said that a lost missing tooth should be replaced by an artificial one. All the participants realized that the use of tobacco and its products was bad for oral and general health similar to the results of the previous study [24].

In the present study, both the preclinical and clinical undergraduate students were adequately oriented toward the importance of maintaining good oral hygiene. The students knew about the importance of regular brushing and maintaining oral hygiene. Almost 86.8% of the preclinical students and 82.6% of clinical students brushed twice a day using toothpaste and brush. Neeraja et al. [25] reported that 74% of the dental students brushed their teeth twice daily. Similarly, a study assessing oral health attitude and behaviour of dental students of four different Asian countries reported that more than 60% of students brushed their teeth twice daily [26]. In the present study, a total of 86.8% of preclinical students and 92.8% of clinical students used fluoridated toothpaste while only 75.4% of clinical students used additional aids to maintain oral hygiene. This number was less in preclinical students where only 72.6% of preclinical students used other aids to maintain oral health. Our observations were in agreement with the findings of the previous studies [24, 27]. The preclinical students were more aware of the importance of tongue cleaning. In our study, almost 98% of the participants used tobacco in some form or the other. Balogh et al. [28] reported that students from other nations like Germany (11.30%), Bangladesh (22%), Holland (24%), Norway (24%), Greece (47%), Serbia (43%), Hungary (34%), France (33%), Italy (33%) and Turkey (26%) had lower smoking rate. Conflicting results from other studies might be attributed to religious, social, and demographic differences.

While the results of the present study offer valuable insight into our understanding of differences in oral health knowledge, attitudes, and behavior among preclinical and clinical dental students it is important to recognize certain limitations. The findings of this study may not be true representative of other dental institutes in the country, and caution should be exercised in extending their applicability to other dental students in India. Future studies should try to address this problem and include multiple institutes and a larger sample size for better external validity.

## Conclusions

Within the limitations of the study, it could be concluded that clinical dental students of the institute showed a marginally higher KAP regarding oral health than preclinical students. This might reveal an ineffective transition of the students from the preclinical to the clinical stage. On intergender comparison, the females were better oriented than males towards oral health.

The students need to have adequate knowledge and attitude about oral health so that they can fulfill their role as oral health educators. For a smooth and successful preclinical-clinical transition, a review and modification of the academic curriculum might be warranted. Moreover, counseling students to develop empathy could also help them to be good oral health educators. Clinical assessment of the students might help to explore the status of oral health in both groups.
